# Progress in General Surgery

**Published:** 1902-05-24

**Authors:** 


### Progress in General Surgery.

(*Continued from page* 119.)

**Lower Extremity.**—Dr. M. Tench [15] briefly relates an interesting case of complete recovery after very extensive injuries. A labourer, age 55, was run over by a threshing machine. Both iliac bones were broken without injury to bladder or urethra, although the rectum was torn and extruded through a large perineal rent. A large hæmatoma extended from the buttocks to the thighs. Several ribs on both sides were broken, so that the sternum protruded and was movable with crepitus. Emphysema existed over the whole trunk and both arms to the wrist. The left clavicle was comminuted. Although extensive sloughing of the back and buttocks ensued, exposing the sacrum, the patient left the infirmary six months after the accident, walking without limping, and later on contemplated resuming his work. If Royal Whitman's statement be accepted, that injuries of the neck of the femur in children under ten are fractures, and that it is not until the age of puberty that the epiphysis is sufficient of an entity to be separated, then, according to Mr. H. L. Barnard,[16] there is very interesting age-classification of injuries to the neck of the femur: (1) Fracture of the neck (Whitman) 2–10 years; (2) separation of epiphysis, 15–18 years; (3) coxa vara (traumatic), 2–18 years; (4) extracapsular fracture of the neck, about 30–40 years; (5) intracapsular fracture, 50–70 years. He gives the signs of fracture of the neck of the femur in children as follow: Leg actually shorter than its fellow; the trochanter is raised and prominent, and the limb is everted. The loss of function is usually evanescent. In a day or two the child is able to walk, with a limp, and the only signs then are pain on manipulation and a little limitation of movement, especially on adduction, flexion, and inward rotation. The treatment is rest and extension for at least a month. This injury, like separation of the upper epiphysis of the femur, may be followed by coxa vara traumatica. Cases of coxa vara traumatica vary from 2 to 18 years of age in their time of onset. The injuries which lead to this subsequent bending of the femoral neck appear to be: (1) Partial fracture of the neck; (2) traumatic ostitis, leading to rarefaction and softening; (3) the increased obliquity of the epiphyseal line will throw a greater proportion of the body weight upon its lower part and less upon its upper. The rate of growth will therefore be slower below than above, and the deformity will tend to become progressively worse. The signs are like the idiopathic variety: Progressive stiffness, pain, and disability in the hip, trochanter prominent and raised, leg shortened, considerable apparent straitening from tilting of pelvis, progression on balls of toes with foot in equinus position, leg adducted and everted. Adduction, inversion, and to a less degree flexion are much limited. Rest and extension should be employed in early cases. Where the deformity is advanced M. Barnard recommends cuneiform osteotomy just below the great trochanter. The adduction of the leg is thereby corrected, whilst the obliquity of the neck is again restored. Two interesting cases of recent traumatic perineal dislocation of the hip joint are described by Mr. H. M. Rigby [17] in one of which death ensued under chloroform and afforded an unique dissection of the hip.

A powerful young man, aged 23, was struck while working in the hold of a ship by a heavy bale on the antero-external aspect of his right femur which was in the abducted position. On admission the leg was flexed to an angle of 30°, abducted to

40° with the sagittal plane, and slightly rotated outwards. There was lengthening of one inch, and the trochanter was buried with distinct flattening and broadening of the buttock. The head of the femur could be made out in the perinæum. Reduction was easily effected, the head first of all slipping into the dorsal position. A long Liston splint was being applied when the patient suddenly stopped breathing and died. The dissection showed the sciatic nerves lying on the posterior wall of a cavity which contained blood and bruised and torn tissue. The gluteus medius, pyriformis, gemellus superior and obturator internus were intact, but the gemellus inferior though untorn was stretched and bruised on its anterior surface. The upper fibres of the tendon of the obturator externus were torn in two, and the fleshy fibres partly turned within the acetabulum, and caught between the edge of the torn capsule and the neck of the femur. The quadratus femoris was torn right across, as if with a knife. The capsule was torn completely through and stripped from the acetabular edge at the lower and inner part, the rent extending from the upper and outer part of the acetabular margin to the upper end of the digital fossa. The cotyloid ligament and edge of the acetabulum were intact. In front much extravasation of blood existed between the insertion of the rectus, vastus externus, hamstrings, and outer surface of the iliacus. The upper fibres of the vastus externus were torn through at their attachment. The lower and inner part of the obturator externus over the region of the cotyloid notch was torn through, and next to the quadratus suffered most damage. A hole with ragged edges of about the size of a halfpenny was seen, evidently caused by the escape of the femoral head during dislocation. The obturator nerve was uninjured. The ligamentum teres was torn through and hanging freely in the joint. The Y-shaped ligament was intact. A case of fracture of the femur just below the trochanter in an infant three months old is reported by Dr. H. A. Wilson[18] as having been produced by a difficult labour. It was a vertex presentation, and forceps had been applied unsuccessfully; version was therefore performed, and considerable force applied upon the femur, the legs being grasped near the knees. The force employed was not that of traction only, but of rotation as well. No fracture or dislocation was made out at the time of delivery. Examination of the seat of fracture showed an enlargement, due to a mass of callus and malposition, the fragments having united at an obtuse angle, the apex pointing forwards. An incision was made, and the exuberant callus chiselled away, the fracture reproduced, and the fragments put in proper position. The delicate bone could not hold a wire suture, therefore a splint was applied, and both legs and trunk put up in plaster of Paris, the legs being slightly abducted. Recovery was uninterrupted. At the end of four weeks the dressings were removed and the result found to be perfect. Dr. Wharton[19] relates an instance of compound fracture of the tibia and fibula in the middle third accompanied with great contusion of the leg and thigh, and a fracture of the internal condyle of the opposite femur. The man, aged 25, had been caught between two cars. The compound fracture of the leg was treated in the usual way with antiseptic dressings and did well. The internal condyle of the femur appeared to be displaced downward into the joint and caused locking of the joint in attempts at extension: this proved to be the case on taking a skiagraph. Three weeks after the injury an incision was made over the inner side of the knee and the internal condyle of the femur found to be separated from the shaft and so twisted that its fractured surface was presenting in the joint and its articular surface turned towards the fractured surface of the femur. The fragment was removed, and a large drainage tube passed into the joint, and the wound closed by sutures. The limb was put up in plaster of Paris in the extended position. The fragment removed consisted of a large portion of the internal condyle measuring $2\frac{1}{2}$ inches long by $1\frac{1}{2}$ inch wide. The patient left the hospital in two months' time, and some months later could walk with a stick and had regained a very fair range of motion in the knee-joint.

[15] Lancet, Feb. 15, 1902, p. 148. [16] Practitioner, Oct., 1901, p. 387. [17] Lancet, Jan. 25, 1902, p. 220. [18] Annals of Surgery, June, 1901, p. 775. [19] Ibid., p. 787.

